# Metabolic Dysfunction-Associated Steatotic Liver Disease and Steatohepatitis (MASLD/MASH) and Cardiovascular Risk: Emerging Therapeutic Strategies at the Interface of Liver and Cardiometabolic Disease

**DOI:** 10.3390/life16101656

**Published:** 2026-10-01

**Authors:** Chrysoula Boutari, Konstantinos Charalampidis, Lazaros Sideras, Emmanouil Sinakos, Ioannis Goulis

**Affiliations:** 4th Department of Internal Medicine, Hippokration General Hospital, Aristotle University of Thessaloniki, 54642 Thessaloniki, Greece

**Keywords:** MASLD, MASH, cardiovascular risk, GLP-1 receptor agonists, dual incretin agonists, FGF 21 analogs, thyroid hormone receptor-β agonists, PPAR agonists

## Abstract

Metabolic dysfunction-associated steatotic liver disease (MASLD) and its progressive form, metabolic dysfunction-associated steatohepatitis (MASH), are increasingly recognized as systemic cardiometabolic disorders rather than isolated liver diseases. Cardiovascular disease (CVD) represents the leading cause of mortality in patients with MASLD/MASH, highlighting the need for therapeutic strategies that address both hepatic and cardiovascular risk. MASLD/MASH and CVD are connected through a network of interdependent mechanisms, including adipose tissue dysfunction, insulin resistance, altered insulin–glucagon signaling, atherogenic dyslipidemia, lipotoxicity, mitochondrial and endoplasmic reticulum stress, innate immune activation, chronic inflammation, endothelial dysfunction, thrombogenic signaling, and dysregulated hepatokine and adipokine pathways. These mechanisms provide potential therapeutic entry points across the liver–adipose tissue–vascular axis. Lifestyle modification and conventional cardiometabolic therapies remain foundational, while several pharmacological agents increasingly target specific components of this network. We summarize the mechanistic rationale and clinical evidence for emerging therapies, including glucagon-like peptide-1 receptor agonists, dual incretin agonists, fibroblast growth factor 21 analogs, thyroid hormone receptor-β agonists, peroxisome proliferator-activated receptor agonists, and agents targeting de novo lipogenesis. We further discuss why residual metabolic, inflammatory, and fibrogenic activity may limit monotherapy and how mechanism-based combination therapy could improve efficacy while mitigating treatment-related metabolic liabilities. Finally, we outline future priorities, including biomarker-guided patient selection, longitudinal noninvasive monitoring, integrated hepatic–cardiovascular trial endpoints, adaptive combination strategies, and development of therapies targeting immune, fibrogenic, and inter-organ signaling pathways.

## 1. Introduction

Metabolic dysfunction-associated steatotic liver disease (MASLD) is defined as accumulation of excessive fat—primarily triglycerides—in the liver in the presence of at least one cardiometabolic risk factor including overweight or obesity, type 2 diabetes mellitus (T2DM), arterial hypertension and/or dyslipidemia in the absence of substantial alcohol consumption and non-metabolic conditions that promote liver steatosis [1]. This term has recently replaced the term non-alcoholic fatty liver disease (NAFLD) and encompasses the same population of patients, as supported by several validation studies [2,3].

MASLD is currently the leading cause of chronic liver disease and its prevalence is increasing worldwide, with an estimated global prevalence of approximately 38% [4,5,6]. Projections suggest a further increase in prevalence by 2040, affecting up to 55% of the global population [7]. A subset of individuals with MASLD may progress to metabolic dysfunction-associated steatohepatitis (MASH), a more severe phenotype that often follows a subclinical course, resulting in advanced liver fibrosis, cirrhosis and hepatocellular carcinoma [1].

In recent years, MASLD is increasingly recognized as a systemic disease. It is considered as a significant and independent risk factor for atherosclerotic cardiovascular disease (ASCVD) and it strongly correlates with the development of T2DM, chronic kidney disease (CKD) and several types of extrahepatic cancers [8,9]. A systematic review and meta-analysis of 5.5 million participants identified MASLD as an independent factor for cardiovascular events with an excessive risk of 45% in individuals with MASLD, a risk that increases further with advancing fibrosis stage [10]. Its heavy metabolic burden is further highlighted by the fact that cardiovascular disease (CVD) represents the leading cause of mortality in this population [11].

Considering CVD remains the primary cause of death in Western populations and in the context of the rising pooled prevalence of T2DM and obesity [11], it becomes evident that early patient identification and risk stratification of MASLD are of critical importance, given the massive socioeconomic burden and implications for public health [5].

Recently, several agents have emerged as promising therapeutic strategies for MASLD. These therapies target either metabolic pathways, achieving benefit primarily from body weight reduction and improved insulin sensitivity or liver-specific inflammation and fibrosis pathways, attenuating or resolving liver disease progression [12,13].

Given the close relationship between MASLD and CVD, they are tightly linked through overlapping biological pathways including insulin resistance (IR), atherogenic dyslipidemia, chronic inflammation, oxidative stress, endothelial dysfunction and a prothrombotic state [14]. Thus, therapies that target both metabolic and liver-specific processes are poised to confer the highest benefit for these patients.

Semaglutide, a glucagon-like peptide 1 receptor agonist (GLP-1 RA) already approved for the treatment of T2DM and/or obesity, and resmetirom, a selective thyroid hormone receptor-β (THR-β) agonist, have now become the first pharmacological agents specifically authorized for MASH with moderate-to-advanced fibrosis (F2–F3). Resmetirom received accelerated approval from the U.S Food and Drug Administration (FDA) in March 2024, followed by a conditional marketing authorization in the European Union (EU) in August 2025 [15,16]. Semaglutide subsequently received FDA accelerated approval in August 2025 and a conditional marketing authorization in the EU in March 2026 [17,18]. Both agents are currently authorized for adults with non-cirrhotic MASH and moderate-to-advanced fibrosis (F2–F3). Approval was granted following clinical trials demonstrating significant histological benefits, including resolution of steatohepatitis and/or improvement in liver fibrosis by at least one stage [19,20,21]. A large pipeline of agents with different mechanisms of action are currently being studied in phase II and III trials [12,22,23]. Moreover, several trials are ongoing, investigating those agents in long-term hepatic and cardiovascular outcomes.

This review aims to explore the interplay between MASLD/MASH and cardiovascular risk as well as highlight current and emerging therapeutic strategies in the context of liver and cardiometabolic disease.

## 2. Mechanistic Links Between MASLD/MASH and Cardiovascular Disease

MASLD and MASH are increasingly understood as disorders of metabolic and immune communication across the liver, adipose tissue, skeletal muscle, gut, and cardiovascular system. CVD is the leading cause of mortality in this population, and the risk of major adverse cardiovascular events (MACE) increases with more advanced liver disease, particularly fibrosis stage [24,25,26,27]. Rather than representing parallel consequences of obesity and diabetes alone, hepatic and vascular injury appear to be linked by a network of interacting metabolic, inflammatory, redox, endocrine, and thrombo-inflammatory pathways. Importantly, the contribution of these pathways is likely to vary according to disease stage and phenotype, which may explain the heterogeneous response to currently available therapies [28,29].

A central initiating event is impaired metabolic flexibility, driven by adipose tissue dysfunction and insulin resistance (IR). In insulin-resistant adipose tissue, failure of insulin to suppress lipolysis increases circulating non-esterified fatty acids (NEFAs), while visceral adipose tissue may provide a particularly important portal source of fatty acids and inflammatory mediators [28,30]. The liver receives this excess substrate together with glucose, fructose, and other nutrients and partitions it among oxidation, triglyceride storage, very low-density lipoprotein (VLDL) secretion, and de novo lipogenesis (DNL). Although triglyceride storage in hepatocytes can initially function as a relatively inert mechanism for buffering excess fatty acids, accumulation of bioactive lipid intermediates such as diacylglycerols (DAGs) and ceramides is more closely linked to cellular stress and IR [31,32].

The molecular basis of hepatic IR is therefore more complex than impaired glucose disposal alone. Hepatic DAG accumulation can activate protein kinase C-ε, disrupting insulin receptor/IRS signaling and downstream phosphatidylinositol 3-kinase–Akt pathways. Ceramides can further inhibit Akt signaling and promote mitochondrial dysfunction and apoptosis. This creates a paradoxical state in which insulin fails to adequately suppress hepatic glucose production while insulin- and nutrient-responsive lipogenic pathways remain active, favoring continued DNL and hepatic lipid accumulation. At the systemic level, persistent hyperglycemia and NEFA exposure promote ectopic lipid deposition in skeletal muscle and myocardium and contribute to the atherogenic dyslipidemia characterized by increased triglyceride-rich lipoproteins, VLDL remnants and reduced HDL cholesterol [32,33]. Thus, insulin resistance acts not simply as a risk factor but as a mechanistic bridge linking hepatic steatosis, diabetes, dyslipidemia, and vascular disease.

Lipotoxicity then converts metabolic overload into cellular injury. Excess fatty acid flux can exceed the capacity of mitochondrial β-oxidation and adaptive lipid storage, resulting in mitochondrial redox imbalance, reactive oxygen species (ROS) generation, endoplasmic reticulum stress, and activation of stress-responsive pathways such as JNK and NF-κB. Damaged hepatocytes release damage-associated molecular patterns that activate Kupffer cells and recruit monocyte-derived macrophages. Toll-like receptor and NLRP3 inflammasome signaling can amplify production of cytokines and chemokines, including tumor necrosis factor-α (TNF-α) and interleukin (IL)-1β, thereby sustaining hepatic inflammation [29,32,34]. Importantly, macrophage responses are heterogeneous: lipid-associated and reparative macrophage populations may participate in efferocytosis, lipid handling, and tissue remodeling, suggesting that future therapies should aim not only to suppress inflammation but also to restore appropriate resolution and repair responses [32].

Fibrogenesis represents a further mechanistic convergence point between metabolic injury and disease progression. Persistent hepatocyte stress and macrophage-derived mediators activate hepatic stellate cells (HSC), which undergo myofibroblast-like transformation and produce extracellular matrix. Transforming growth factor-β, oxidative stress, inflammatory signaling, and altered metabolic cues contribute to this process, while repeated cycles of hepatocyte injury and repair sustain extracellular matrix deposition [26,27,32]. Fibrosis should therefore be viewed as a dynamic, potentially modifiable process rather than a passive consequence of steatohepatitis. The stronger association of fibrosis stage with clinical outcomes also emphasizes that therapeutic strategies capable of interrupting the transition from metabolic injury to persistent fibrogenesis may have greater prognostic relevance than approaches that merely reduce hepatic fat [35].

The liver–adipose–gut axis adds additional layers of regulation. Adipose-derived cytokines and adipokines modify hepatic insulin sensitivity and immune activation, while altered hepatokine signaling may influence systemic glucose and lipid metabolism. At the same time, intestinal barrier dysfunction, changes in microbial composition, and altered bile-acid signaling can increase exposure to microbial products and modify farnesoid X receptor (FXR) and other metabolically relevant pathways. These signals can reinforce hepatic innate immune activation and fibrogenesis, although human evidence for a causal role of gut dysbiosis remains less definitive than experimental data [32,36,37,38,39]. This complexity is therapeutically important because it suggests that successful treatment may require simultaneous correction of upstream metabolic drivers and downstream hepatic injury rather than targeting steatosis in isolation.

**Figure 1:**
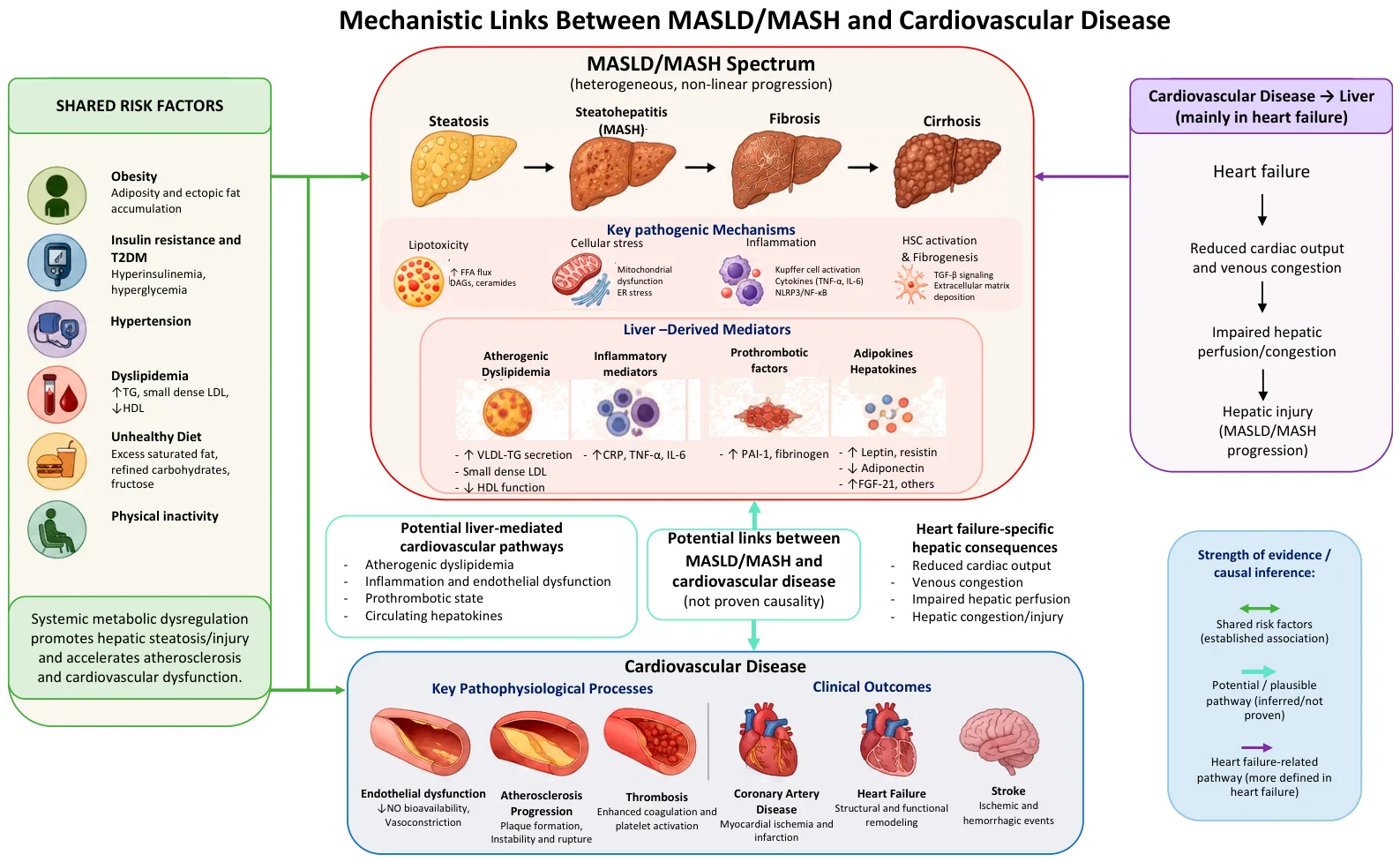
Mechanistic Links Between MASLD/MASH and Cardiovascular Disease. Shared metabolic risk factors underlie both MASLD/MASH and cardiovascular disease, while potential liver-mediated pathways may contribute to cardiovascular risk. The reverse pathway is most clearly recognized in heart failure, where reduced cardiac output and venous congestion can promote hepatic injury. MASLD: Metabolic Dysfunction-Associated Steatotic Liver Disease; MASH: Metabolic dysfunction-Associated Steatohepatitis; DAG: diacylglycerol; ER: endoplasmic reticulum; FFA: free fatty acids; FGF-21: fibroblast growth factor-21; T2DM: Type 2 Diabetes Mellitus; TG: Triglycerides; LDL: Low-Density Lipoprotein; HDL: High-Density Lipoprotein; VLDL: Very Low-Density Lipoprotein; CRP: C-Reactive Protein; TNF-α: Tumor Necrosis Factor-α; IL-6: Interleukin-6; PAI-1: Plasminogen Activator Inhibitor-1.

The same network can promote cardiovascular injury [Figure 1]. Hepatic overproduction of triglyceride-rich lipoproteins and impaired clearance of remnant particles increase the pool of atherogenic lipoproteins reaching the arterial wall. Systemic inflammation promotes endothelial activation and leukocyte recruitment, while ROS reduce nitric oxide (NO) bioavailability and increase oxidative modification of lipoproteins [37,38,40,41,42]. In parallel, increased circulating adhesion molecules, von Willebrand factor, and plasminogen activator inhibitor-1 (PAI-1) may contribute to a prothrombotic milieu and plaque instability [40]. These mechanisms provide a plausible biological connection between hepatic disease activity and ASCVD [43]; however, the observational association between fibrosis and cardiovascular events should not be interpreted as proof that fibrosis itself is directly causal. Rather, fibrosis may function as an integrative marker of cumulative metabolic, inflammatory, and vascular injury [24,25,26,27].

Taken together, MASLD/MASH can be conceptualized as a multi-organ disease in which metabolic substrate overflow, altered insulin–glucagon signaling, lipotoxicity, mitochondrial and endoplasmic reticulum stress, innate immune activation, fibrogenesis, and vascular dysfunction form interconnected feed-forward loops [Figure 1]. This framework has direct therapeutic implications: interventions acting upstream on obesity, IR and substrate flux may prevent or attenuate hepatic injury, whereas liver-directed agents may be required to suppress persistent inflammation and fibrosis once these processes become established. The most effective future strategies are therefore likely to be mechanism-based, stage-specific, and capable of simultaneously modifying hepatic and cardiovascular risk [32,37,44].

To avoid conflating different levels of evidence, cardiovascular effects discussed below are classified as established cardiovascular outcome benefit when supported by randomized cardiovascular outcome trials; demonstrated cardiovascular safety when outcome trials primarily establish noninferiority or absence of excess cardiovascular risk; favorable cardiovascular risk-factor effects when improvements are limited to parameters such as LDL-C, triglycerides, body weight, blood pressure, or glycemic control; and mechanistic or preclinical cardiovascular rationale when evidence derives primarily from experimental, molecular, or biomarker studies.

## 3. Established Therapeutic Approaches and Their Cardiovascular Effects

### 3.1. Lifestyle and Behavioral Interventions

Given the close relationship between MASLD and CVD, established therapeutic strategies, such as lifestyle modifications and conventional cardiometabolic therapies, play a fundamental role in modifying both hepatic and vascular outcomes [42,45]. Lifestyle modification remains the cornerstone of the therapeutic approach in MASLD patients and also exerts beneficial effects on cardiovascular health.

Weight loss, achieved through diet and physical activity, is related with improvement in hepatic steatosis, inflammation, and even in fibrosis [46,47,48]. It has been suggested that the weight loss of 7–10% leads to histological improvement in steatohepatitis, and an even greater weight reduction may result in fibrosis improvement [46]. Of note, these changes are accompanied by improvements in metabolic parameters like insulin sensitivity, lipid profile, and hypertension, contributing to a significant reduction in cardiovascular risk [42,45]. As regards physical exercise, even in the absence of significant weight reduction, it improves hepatic insulin sensitivity and reduces circulating inflammatory markers. It also increases endothelial NO bioavailability and reduces circulating inflammatory markers [49,50].

Moreover, the dietary pattern exerts a strong effect on metabolic syndrome components and on the hepatic fat content, thus exerting benefits on atherosclerotic cardiovascular risk. Diets rich in unsaturated fatty acids, fiber, and antioxidants, such as the Mediterranean diet, have been suggested to reduce liver fat content, systemic inflammation, and atherosclerosis progression [48,51,52]. To the contrary, excessive consumption of refined carbohydrates, trans and saturated fats, and added sugar or fructose promotes DNL and, subsequently, both hepatic and vascular injury [53].

### 3.2. Conventional Cardiometabolic Therapies

The conventional cardiometabolic therapies seem to provide substantial hepatic and cardiovascular benefits. Statins are the cornerstone of LDL-C lowering and ASCVD prevention and have a well-established cardiovascular outcome benefit in appropriately selected patients. They are generally safe in patients with MASLD and chronic liver disease and should not be withheld solely because of the presence of steatotic liver disease. Observational and mechanistic studies have suggested that statins may also be associated with favorable effects on hepatic steatosis, inflammation, or disease progression; however, evidence supporting a direct disease-modifying effect on MASH histology or fibrosis remains substantially less definitive than the evidence supporting their cardiovascular benefit. Accordingly, statins should be prescribed primarily according to established cardiovascular risk and lipid-lowering indications rather than as disease-specific therapy for MASH [54,55,56].

Among antihypertensive agents, inhibitors of the renin–angiotensin–aldosterone system (RAAS) have attracted interest because of their potential effects on liver fibrosis and vascular remodeling [57]. Experimental studies suggest that RAAS activation promotes HSC activation and fibrogenesis, raising the possibility that RAAS blockade may attenuate these processes. Although RAAS inhibitors remain the cornerstone of antihypertensive therapy when clinically indicated, current clinical evidence does not support their use specifically for the treatment of MASLD or MASH. Nevertheless, their established cardiovascular and renal benefits make them an important therapeutic option in patients with MASLD who have appropriate indications for RAAS inhibition [57,58].

The prevalence of MASLD in patients with type 2 diabetes mellitus (T2DM) is up to 60–75% and a more rapid progression of MASLD towards its more advanced stages is observed [59]. Moreover, this group of patients is at markedly increased cardiovascular risk [59]. For these reasons, glucose-lowering therapies are essential for cardiovascular risk management in patients with MASLD and coexisting T2DM. Metformin improves insulin sensitivity and, in parallel, reduces gluconeogenesis and liponeogenesis in the liver and inhibits systemic inflammation. It has been shown that it reduces hepatic steatosis and transaminase levels without a significant effect on fibrosis and resolution of MASH [55,60].

Sodium-Glucose Cotransporter-2 Inhibitors (SGLT2i) exert favorable cardiovascular effects and metabolic benefits. These agents enhance urinary glucose excretion, improve glycemic control, lower blood pressure, and cause modest weight loss, all of which alleviate hepatic steatosis and CVD [61]. It has also been shown that SGLT2i reduce liver fat content and transaminase levels in patients with MASLD/MASH by improving insulin sensitivity, decreasing glucotoxicity, and modifying hepatic lipid flux [61,62]. Interestingly, large trials have highlighted important cardiovascular effects, such as significant reductions in heart failure hospitalization and MACEs with these glucose-lowering agents [63].

Incretin-based therapies, including Glucagon-like peptide-1 Receptor Agonists (GLP-1 RAs), improve glycemic control through glucose-dependent insulin secretion and cause notable weight reduction. Furthermore, their beneficial effects on hepatic insulin sensitivity, lipid metabolism, and inflammatory signaling lead to histological improvement in steatosis and steatohepatitis [64]. As for their cardiovascular benefits, GLP-1 RAs improve endothelial function, reduce systemic inflammation, and contribute to blood pressure and lipid profile control and, consequently, they result in markable reductions in MACEs [65].

Vitamin E has been evaluated as an antioxidant therapy targeting oxidative stress, a relevant mechanism in MASH pathogenesis. In the PIVENS trial, treatment with natural vitamin E (rrr-α-tocopherol, 800 IU/day) for 96 weeks resulted in significant histological improvement in non-diabetic adults with biopsy-proven NASH compared with placebo [66]. Vitamin E improved steatosis, lobular inflammation, and hepatocellular ballooning, although a significant improvement in fibrosis was not demonstrated.

Accordingly, AASLD guidance supports consideration of vitamin E in selected non-diabetic adults with NASH, particularly when the potential benefits and risks have been carefully discussed [67]. However, vitamin E has not demonstrated cardiovascular outcome benefit and should not be considered a cardiovascular risk-reducing therapy. Long-term use of high-dose vitamin E also requires caution because of historical concerns regarding potential adverse effects, including conflicting findings from meta-analyses regarding all-cause mortality, as well as other reported safety signals [66]. Therefore, its use should be individualized, and the uncertain long-term safety profile should be balanced against its potential histological benefit.

### 3.3. Bariatric and Metabolic Surgery

Bariatric or metabolic surgery represents an established therapeutic option for appropriately selected individuals with obesity and has important implications for the management of MASLD/MASH. By inducing substantial and sustained weight loss, metabolic surgery can improve several key drivers of liver disease, including insulin resistance, adipose tissue dysfunction, dyslipidemia, systemic inflammation, and ectopic fat accumulation [68]. Current European clinical practice guidelines recommend considering bariatric surgery in adults with non-cirrhotic MASLD and an approved indication for surgery because of its long-term beneficial effects on liver disease, remission of T2DM, and improvement in cardiometabolic risk factors [1].

Evidence from longitudinal studies and systematic reviews indicates that bariatric procedures can induce marked improvements in hepatic steatosis and steatohepatitis and, in a substantial proportion of patients, regression or improvement of fibrosis. Roux-en-Y gastric bypass and sleeve gastrectomy are among the most extensively studied procedures, although the magnitude of hepatic benefit appears to be closely related to the degree and durability of weight loss [67,68].

Beyond hepatic effects, bariatric surgery provides broad cardiometabolic benefits through sustained reductions in body weight and improvements in glycemic control, blood pressure, and atherogenic dyslipidemia. Observational studies and a recent meta-analysis in patients with MASLD have suggested a lower incidence of cardiovascular events following bariatric surgery compared with non-surgical management [69]. However, these data should be interpreted cautiously because the available evidence is derived predominantly from observational cohorts rather than dedicated randomized cardiovascular outcome trials in patients with MASH. Thus, bariatric/metabolic surgery has strong evidence for improving obesity-related metabolic disease and liver histology, while its apparent cardiovascular benefits in MASLD are supported mainly by observational outcome data.

Appropriate patient selection remains essential. Bariatric surgery should be considered within established obesity-treatment criteria and requires individualized assessment of liver disease severity. In patients with compensated advanced chronic liver disease or cirrhosis, careful multidisciplinary evaluation is required, particularly with regard to portal hypertension and procedure-specific risks [1,67].

## 4. Emerging Therapeutic Options

### 4.1. Peroxisome Proliferator-Activated Receptors (PPARs)

PPARs are ligand-activated transcription factors belonging to the nuclear receptor superfamily. They regulate genes involved in cellular differentiation and metabolic processes, like lipid and glucose homeostasis [70,71]. There are three isoforms, PPARα, PPARδ, and PPARγ, which have different expression patterns. PPARα is the predominant isoform in the liver. It is involved in energy homeostasis regulation and downregulates plasma triglyceride levels. PPARγenhances insulin sensitization and glucose metabolism, while PPARβ promotes fatty acid metabolism [70,71].

Lanifibranor, a pan-PPAR agonist, activates all three PPAR isoforms and, thus, causes PPARα-regulated inhibition of hepatic steatosis, PPARδ-regulated reduction in hepatic macrophage infiltration, and PPARγ-regulated reduction in HSCs activation [72,73]. Consequently, lanifibranor is supposed to impact fibrogenesis through the regulation of inflammatory responses and by inhibition of the HSCs activation. In the phase 2b NATiVE trial (NCT03008070) 24 weeks of 800 mg and 1200 mg lanifibranor treatment resulted in a dose-dependent resolution of MASH without worsening of fibrosis (22% vs. 39% vs. 49% [*p* < 0.001] of patients on placebo, 800 mg, and 1200 mg lanifibranor, respectively) [74]. Moreover, an improvement in fibrosis of at least one stage without worsening of MASH was observed in 29% vs. 34% vs. 48% of subjects receiving those doses (*p* = 0.011). A phase 3 trial is underway, and its results are expected in the second half of 2026 (NCT04849728).

From a cardiovascular perspective, PPAR agonists have several potentially favorable metabolic and vascular effects, including improvements in insulin sensitivity, triglyceride concentrations, and inflammatory signaling [75]. Experimental studies have also suggested beneficial effects on endothelial function and atherosclerotic processes [76,77]. These findings provide a mechanistic cardiovascular rationale, but they should not be interpreted as evidence of cardiovascular event reduction with lanifibranor or other investigational PPAR agonists. Moreover, PPARs direct cardiac energy metabolism in cardiomyocytes and thus, they positively affect heart failure and diabetic cardiomyopathy [76,77]. Nevertheless, PPAR agonists, particularly those with PPARγ activity, are associated with fluid retention and peripheral edema, which may limit their use in patients at risk of heart failure. Therefore, careful patient selection and monitoring are warranted [78].

### 4.2. Incretin-Based Therapies

Incretin-based therapies have emerged as one of the most promising pharmacologic approaches for MASLD, since they target the metabolic disorders that drive both hepatic injury and CVD. The glucagon-like peptide-1 receptor agonists (GLP-1 RAs), the dual GLP-1/glucose-dependent insulinotropic polypeptide (GIP) RAs, and the investigational triple agonists exert positive effects on weight loss, insulin resistance, dyslipidemia, and systemic inflammation.

GLP-1 RAs, including liraglutide and semaglutide, exert beneficial effects on hepatic steatosis and even steatohepatitis. These pharmaceutical agents reduce energy intake, promote substantial weight loss, improve insulin sensitivity, and downregulate postprandial glucose and lipotoxic substrate delivery to the liver. The interim results of the ongoing phase 3 ESSENCE trial (NCT04822181) showed that in patients with MASH and moderate or advanced liver fibrosis, once weekly semaglutide 2.4 mg improved liver histologic results (MASH resolution and liver scarring) [79]. Based on these results, this agent has received accelerated approval from FDA in August 2025 and EU conditional marketing authorization in March 2026 for adults with non-cirrhotic MASH and F2–F3 fibrosis [17,18]. Moreover, liraglutide improved hepatic steatosis and metabolic parameters in patients with MASLD [80].

Beyond the traditional GLP-1 RAs, multi-agonists represent a promising class of therapies for patients with MASLD and MASH. Tirzepatide combines GIP and GLP-1 receptor agonism and results in even greater reductions in body weight compared to GLP-1 RAs alone. Its therapeutic potential was highlighted in the SYNERGY-NASH trial (NCT04166773), in which tirzepatide significantly increased rates of MASH resolution without worsening fibrosis, compared with placebo in patients with biopsy-confirmed hepatic fibrosis stage F2-F3 [81]. Moreover, the SYNERGY-OUTCOMES study (NCT07165028), an active, ongoing phase 3 study, is evaluating whether the once-weekly medications tirzepatide and retatrutide can prevent major adverse liver outcomes (MALO) in patients with high-risk MASLD.

Another promising multi-agonist is survodutide (BI 456906), a dual glucagon and GLP-1 receptor agonist designed to combine the metabolic benefits of GLP-1 receptor activation with the increased energy expenditure mediated by glucagon receptor agonism. In a phase 2 randomized trial involving patients with biopsy-confirmed MASH and fibrosis stages F1–F3, survodutide significantly increased MASH resolution and improved hepatic fibrosis, while also reducing liver fat content, body weight, and biomarkers of hepatic inflammation [82]. Beyond its hepatic effects, the addition of glucagon receptor agonism may confer cardiometabolic benefits through mechanisms extending beyond weight loss, including enhanced lipolysis, reductions in visceral and epicardial adipose tissue, attenuation of systemic inflammation, and favorable effects on cardiac remodeling and fibrosis [83]. Preclinical studies have also suggested potential renal and metabolic benefits associated with glucagon receptor activation [84]. Although these mechanistic findings support a mechanistic and favorable cardiometabolic rationale, whether survodutide reduces cardiovascular events remains unknown. The ongoing phase III SYNCHRONIZE-CVOT trial is currently evaluating whether survodutide reduces major adverse cardiovascular events in adults with overweight or obesity and established cardiovascular disease, providing the first dedicated assessment of its cardiovascular efficacy (NCT06077864).

In addition to weight-mediated effects and MASH improvement, GLP-1-based therapies exert vascular and anti-inflammatory actions [85,86]. They have been associated with improvements in endothelial function, blood pressure, postprandial lipemia, oxidative stress, and systemic inflammatory burden, all of which are closely relevant to cardiovascular risk reduction. Importantly, cardiovascular outcome trials have shown that GLP-1 RAs reduce MACEs in high-risk populations [85,86].

GLP-1-based therapies have demonstrated cardiovascular benefits in selected high-risk populations, but the evidence differs among individual agents and clinical settings [87]. Specifically, the LEADER (Liraglutide Effect and Action in Diabetes: Evaluation of Cardiovascular Outcome Results) trial showed that in 9000 high risk patients with T2DM liraglutide reduced the risk of MACE by 13% [65]. Similarly, the SUSTAIN-6 (Trial to Evaluate Cardiovascular and Other Long-term Outcomes with Semaglutide in Subjects with Type 2 Diabetes) trial showed that semaglutide reduced the risk for cardiovascular death, nonfatal myocardial infarction (MI) or nonfatal stroke in patients with T2DM and high cardiovascular risk receiving semaglutide, compared with those receiving placebo [88]. These findings represent established cardiovascular outcome benefits in the populations studied, but they should not automatically be extrapolated to patients with MASH without diabetes or without established ASCVD.

Tirzepatide requires particular distinction. In SURPASS-CVOT, which enrolled patients with T2DM and established ASCVD, tirzepatide was noninferior to dulaglutide for the composite of cardiovascular death, myocardial infarction, or stroke (hazard ratio 0.92, 95.3% CI 0.83–1.01; *p* = 0.003 for noninferiority), but superiority was not demonstrated (*p* = 0.09) [89]. Thus, the current evidence supports cardiovascular safety/noninferiority relative to dulaglutide, rather than a demonstrated reduction in cardiovascular events compared with an established cardioprotective GLP-1 receptor agonist. Moreover, cardiovascular outcome data generated in populations with T2DM, obesity, or established ASCVD should not be interpreted as direct evidence of cardiovascular event reduction specifically in patients with MASH.

### 4.3. Farnesoid X Receptor (FXR) Agonists and Bile Acid Signaling Modulators

Bile acids are not only essential for lipid absorption but also function as signaling molecules involved in the regulation of hepatic lipid handling, glucose metabolism, inflammation, and systemic energy homeostasis. These effects are mediated primarily through the nuclear receptor farnesoid X receptor (FXR) and the membrane receptor Takeda G-protein-coupled receptor 5 (TGR5) [90]. Alterations in bile acid homeostasis and signaling have been implicated in MASLD/MASH pathogenesis, including disturbances in hepatic and intestinal FXR signaling, fibroblast growth factor 19 (FGF19)-mediated feedback mechanisms, and bile acid metabolism [91].

FXR activation represents a biologically attractive therapeutic strategy because it can influence several pathways relevant to MASH. Activation of FXR suppresses hepatic bile acid synthesis, modulates de novo lipogenesis and lipid metabolism, and may improve insulin sensitivity. FXR signaling may additionally exert anti-inflammatory and antifibrotic effects through modulation of hepatocellular stress responses, hepatic stellate-cell activation, and inflammatory signaling pathways [92,93]. However, the translation of these mechanistic effects into sustained clinical benefit has proven challenging.

Among FXR-targeted therapies, obeticholic acid (OCA) has been the most extensively studied. Earlier clinical studies demonstrated improvements in biochemical and histological markers of liver injury, and the phase III REGENERATE trial provided evidence of fibrosis improvement without worsening of steatohepatitis in patients with pre-cirrhotic NASH/MASH [94,95,96]. Nevertheless, the effect on MASH resolution was less compelling, and the overall benefit–risk balance was complicated by dose-dependent pruritus and unfavorable effects on circulating lipoproteins, including increases in LDL-C and reductions in HDL-C [96,97]. These adverse effects are particularly relevant in a population characterized by a high burden of obesity, T2DM, dyslipidemia, and cardiovascular disease. In addition, regulatory concerns regarding the overall benefit–risk profile and the need for confirmation of long-term clinical benefit ultimately prevented approval of OCA for MASH [97].

The limitations of first-generation FXR agonism stimulated the development of next-generation agents, including non-steroidal FXR agonists such as tropifexor and cilofexor. Early-phase studies have demonstrated reductions in hepatic fat content and improvements in liver enzymes and bile-acid-related biomarkers [98,99]. However, robust and reproducible histological efficacy, long-term safety, and clinically meaningful liver outcomes remain to be established. Moreover, favorable metabolic or lipid effects observed with individual agents should not be interpreted as evidence of demonstrated cardiovascular outcome benefit.

Overall, FXR agonism remains a biologically compelling approach for MASH because of its potential effects on metabolic, inflammatory, and fibrotic pathways. However, the clinical development of this class illustrates the challenges of translating improvements in histological or surrogate endpoints into an acceptable long-term benefit–risk profile. Future bile acid pathway therapies may therefore need to combine hepatic efficacy with improved tolerability and a neutral or favorable cardiometabolic profile.

### 4.4. FGF21 Analogs

FGF21 is a stress hormone secreted by the liver and adipose tissue with pleiotropic metabolic effects [100]. In adipose tissue, FGF21 enhances glucose uptake and adiponectin secretion and promotes lipid catabolism and lipoprotein lipase activation [101]. Three FGF21-based agents are currently being studied in phase 3 trials in MASH with fibrosis F2 or F3, (efruxifermin in the SYNCHRONY Histology trial [NCT06215716], pegozafermin in the ENLIGHTEN-fibrosis trial [NCT06318169], and efimosfermin alfa in the ZENITH-1 trial [NCT07221227]). Efruxifermin is a subcutaneously administered IgG Fc-FGF21 fusion protein. It resulted in at least a one-stage improvement in liver fibrosis, with no worsening of MASH, in 41% of patients on 50 mg and 39% on 28 mg, compared with 20% in the placebo group in the HARMONY study [102]. Pegozaferminis another subcutaneously administered agent. It is a long-acting glycopegylated recombinant FGF21 analog administered in doses of 15 mg and 30 mg weekly or 44 mg every two weeks. This drug was studied in the phase 2b ENLIVEN study in patients with MASH and F2 or F3 fibrosis [103]. Pegozafermin showed statistically significant fibrosis improvement without worsening of MASH in 22%, 26%, and 27% of patients receiving the 15 mg, 30 mg, and 44 mg regimens, respectively, compared with 7% of those receiving placebo. These findings indicate a numerical improvement in the prespecified fibrosis endpoint; however, the magnitude and statistical significance of the effect varied according to dose and should not be interpreted as evidence of a uniformly statistically significant antifibrotic effect across all treatment groups. Efimosfermin alfa is a monthly subcutaneously administered FGF21 analog. In a phase 2 study involving patients with MASH and fibrosis, efimosfermin alfa was associated with improvement in fibrosis without worsening of MASH in 45.2% of treated patients compared with 20.6% of those receiving placebo [104]. Similarly, MASH resolution without worsening of fibrosis was achieved by 67.7% of patients in the efimosfermin alfa group, compared with 29.4% of those on placebo [104]. Based on these results, efimosfermin alfa has advanced into the ongoing phase 3 ZENITH-1 (NCT07221227) and ZENITH-2 (NCT07221188) trials in MASH with F2 or F3 fibrosis.

FGF21 analogs, including pegbelfermin and efruxifermin, exert favorable systemic metabolic effects that could be relevant to cardiovascular risk. In a phase 2 randomized trial, pegbelfermin resulted in beneficial changes in lipid profiles including reductions in triglycerides and increases in HDL cholesterol, suggesting a favorable impact on atherogenic dyslipidemia [105]. However, longer-term data is needed to establish its impact on cardiovascular outcomes. Efruxifermin has demonstrated robust improvements in lipidemic profile with reductions in triglycerides, non-HDL cholesterol, and apolipoprotein B, as well as improvements in insulin sensitivity and glycemic control [106,107]. Additionally, efruxifermin has been associated with modest weight loss and increased energy expenditure which may translate into improved cardiometabolic milieu [107,108]. These findings constitute favorable cardiovascular risk-factor effects, rather than demonstrated cardiovascular outcome benefit. Although such changes provide a rationale for potential cardiovascular benefit, dedicated cardiovascular outcome data for FGF21 analogues in patients with MASLD/MASH are currently lacking.

### 4.5. Thyroid Hormone Receptor-β (THR-β) Agonists

Thyroid hormone signaling plays a central role in the regulation of hepatic lipid metabolism, energy expenditure, and systemic metabolic homeostasis. Activation of the THR-β isoform, which is predominantly expressed in the liver, enhances fatty acid oxidation, decreases lipogenesis, and promotes cholesterol clearance [109]. In MASLD/MASH, impaired thyroid hormone signaling and subsequent hepatic hypothyroidism are implicated in the development of dyslipidemia and steatosis providing a strong rationale for selective pharmacologic targeting of THR-β [110]. In March 2024, a small-molecule THR-β agonist, resmetirom, which is administered orally everyday became the first pharmacological therapy to receive FDA approval for adults with non-cirrhotic MASH/NASH and moderate-to-advanced fibrosis (F2–F3), in conjunction with diet and exercise. The approval was granted through the FDA accelerated approval pathway and is contingent on confirmatory evidence of clinical benefit [15]. In the European Union, resmetirom received a conditional marketing authorisation in August 2025 for adults with non-cirrhotic MASH and F2–F3 fibrosis [16]. A phase 3 clinical trial in patients with MASH and hepatic fibrosis showed that resmetirom resulted in MASH resolution without fibrosis worsening in 25.9% of patients receiving the dose of 80 mg and 29.9% of patients receiving 100 mg, compared with 9.7% of those receiving placebo. Moreover, fibrosis improvement by at least one stage with no worsening of MASLD was achieved by 25% of patients receiving resmetirom, compared with 14.2% of those on placebo [111]. Additionally, resmetirom has demonstrated favorable effects on lipid panel; reductions in LDL cholesterol, non-HDL cholesterol, apolipoprotein B, and triglycerides. In MAESTRO-NASH, LDL-C reductions were approximately 13.6–16.3%, depending on dose and assessment. These findings represent a favorable cardiovascular risk-factor effect, particularly given the central role of atherogenic lipoproteins in ASCVD. However, these reductions are below the approximately 30–49% LDL-C reduction generally expected with moderate-intensity statin therapy; therefore, resmetirom should not be described as having lipid-lowering efficacy comparable to moderate-intensity statins [21]. However, whether these lipid changes translate into reductions in cardiovascular events in patients with MASH remains unknown. Importantly, clinical trials have shown that resmetirom is generally well tolerated, with a low incidence of cardiovascular adverse effects, supporting the hypothesis that liver-selective THR-β activation can achieve metabolic benefits without systemic thyrotoxic effects associated with non-selective thyroid hormone therapy [111,112,113]. However, long-term cardiovascular outcome data are still pending [19].

VK2809 is another selective THR-β agonist that has been evaluated in separate clinical studies involving different populations and assessment time points. In NCT02927184, an earlier 12-week study in patients with primary hypercholesterolemia and hepatic steatosis, VK2809 produced substantial relative reductions in liver fat content, with reductions of approximately 50–60%, depending on dose and analysis [114]. These values represent relative changes in liver fat content and should not be interpreted as the proportion of patients responding to treatment. Separately, the VOYAGE study (NCT04173065) evaluated VK2809 in patients with MASH over a longer treatment period.

### 4.6. Agents Targeting DNL

Rates of DNL are elevated in MASLD. DNL is a coordinated metabolic pathway in which cytosolic acetyl-CoA serves as the precursor for fatty-acid synthesis. Citrate-derived acetyl-CoA is generated predominantly through ATP-citrate lyase, whereas acetate can contribute to the cytosolic acetyl-CoA pool through acetyl-CoA synthetase. Acetyl-CoA carboxylase (ACC) catalyzes the first committed step of DNL, converting acetyl-CoA to malonyl-CoA, which subsequently enters fatty-acid synthesis. Pharmacologic inhibition of ACC has emerged as an attractive approach to reduce hepatic lipid accumulation. MK-4075 is a liver-selective ACC1/2 inhibitor that effectively inhibits DNL. It reduced hepatic steatosis by 36% after one month of treatment (NCT01431521) [115]. Nevertheless, it significantly increased serum triglycerides, an effect common to all ACC inhibitors. PF-05221304 is a reversible ACC1/2 inhibitor mainly distributed in the liver. This ACC inhibitor was tested in a phase 2a trial as monotherapy, as well as in combination with the diacylglycerol acyltransferase (DGAT) inhibitor PF-06865571. PF-05221304 resulted in an 82% reduction in DNL in the liver and dose-dependent reductions in liver steatosis by 50–65% were observed [116]. GS-0976 (firsocostat) inhibits ACC via another binding site to PF-05221304 and exerts selective hepatic uptake. In an open-label, non-placebo-controlled study in patients with MASH, GS-0976 resulted in a 22% decrease in DNL with a significant reduction in steatosis and hepatic stiffness after 12-week therapy and similar results were presented in a larger placebo-controlled study [117,118]. However, despite these encouraging reductions in liver fat, the clinical development of ACC inhibitors has been limited by treatment-associated hypertriglyceridemia, a class effect thought to result from altered hepatic lipid metabolism. This adverse metabolic profile raises concerns regarding long-term cardiovascular safety, particularly in a population already at high cardiovascular risk.

Another approach to suppress DNL is inhibition of fatty acid synthase (FASN). Denifanstat (TVB-2640), a selective FASN inhibitor, demonstrated encouraging histological efficacy in phase 2b development. In the FASCINATE-2 trial, denifanstat significantly improved disease activity and MASH resolution compared with placebo and was also associated with a significantly greater proportion of patients achieving at least one-stage fibrosis improvement without worsening of MASH [119]. These findings support FASN inhibition as a promising therapeutic strategy targeting DNL, inflammation, and fibrogenesis. However, confirmatory phase 3 studies, longer-term safety data, and evidence demonstrating improvement in clinically meaningful liver or cardiovascular outcomes remain necessary before its therapeutic role can be established.

DGAT enzymes catalyze the final step in triglyceride synthesis by esterifying diacylglycerol with fatty acids to form triglycerides. Although inhibition of DGAT reduces triglyceride synthesis and hepatic lipid storage, its effects on lipotoxicity are context dependent [120]. Inhibition of DGAT2 in preclinical studies led to reduced expression of genes essential for DNL [121]. The DGAT2 inhibitor ervogastat (PF-0686571) was assessed alone and in combination with the ACC inhibitor clesacostat in the phase 2, 48-week MIRNA study [122]. The primary histological endpoint comprised MASH resolution without worsening of fibrosis, improvement in fibrosis without worsening of MASH, or both. Although the combination of ervogastat with clesacostat demonstrated strong efficacy with respect to the prespecified histological endpoints, ervogastat monotherapy did not meet the composite primary endpoint. This finding should not be interpreted as demonstrating an absence of effect on individual histological components, including MASH resolution. Importantly, the combination of ervogastat and clesacostat was also associated with potentially unfavorable changes in lipid and apolipoprotein profiles [122]. These findings are particularly relevant in patients with MASH, who frequently have substantial underlying cardiovascular risk. Therefore, the metabolic and cardiovascular implications of this combination remain uncertain and require further evaluation. Triglyceride esterification and lipid-droplet formation may represent adaptive mechanisms that sequester potentially toxic fatty-acid and diacylglycerol species; therefore, reducing triglyceride synthesis does not necessarily translate into reduced lipotoxic injury. DGAT inhibition may consequently have both potentially beneficial and adverse metabolic consequences depending on the balance between triglyceride storage, lipid export, oxidation, and accumulation of bioactive lipid intermediates. Further studies are required to better define their long-term metabolic and cardiovascular effects and safety. Ongoing investigations are primarily focusing on combination strategies, including ervogastat in combination with resmetirom or other agents, in an effort to improve efficacy while minimizing metabolic adverse effects [123].

The major clinical trials on emerging therapeutic options targeting MASLD/MASH and cardiovascular risk are described in Table 1.

### 4.7. Combination Therapies

Given the complex and multifactorial pathogenesis of MASH—encompassing hepatic steatosis, inflammation, fibrosis, insulin resistance, and systemic cardiometabolic dysfunction—monotherapy may not adequately address all pathogenic mechanisms in every patient. Combination approaches targeting multiple mechanisms may be required to achieve meaningful improvements in liver histology and cardiometabolic risk and this is an active area of investigation in MASH [124,125].

The purpose of targeting multiple pathways through combination therapy is to provide complimentary or synergistic mechanisms of action to increase therapeutic response and reduce side-effects. A reasonable approach could be to combine drugs that target system metabolic drivers of MASH with liver-directed therapy. Regimens combining GLP-1 RAs, FGF21 analogs, or SGLT2 inhibitors with liver-directed therapies (e.g., THR-β agonists or FXR agonist) aim to address both hepatic pathology and systemic drivers, such as obesity, IR, and dyslipidemia. Alternatively, combination therapy may be used wherein one drug offsets adverse effects of another, such as DGAT2 inhibitors reducing triglyceride elevations induced by ACC inhibitors [126]. Similarly, combining agents with different safety profiles may allow for lower individual drug doses while maintaining efficacy. Several phase 2 combination trials have been completed; however, these studies were limited by study populations, treatment duration, and the absence of definitive clinical outcome data [126,127,128,129,130]. Furthermore, some combinations have not demonstrated meaningful efficacy beyond that achieved with monotherapy. For example, semaglutide combined with the FXR agonist cilofexor and/or the ACC inhibitor firsocostat improved liver fat content and metabolic parameters but failed to produce significant improvements in fibrosis compared with semaglutide alone in patients with MASH [131,132]. More recently, although the combination of semaglutide and the amylin analogue cagrilinitide has shown remarkable weight-loss efficacy in obesity [133,134], its role in MASH remains under investigation, and evidence supporting additional histological benefit is currently lacking. Thus, several ongoing clinical trials are evaluating semaglutide in combination with liver-directed therapies in an effort to simultaneously address systemic metabolic dysfunction and intrahepatic disease mechanisms. Combinations under investigation include semaglutide with resmetirom, based on their complementary mechanisms as well as combinations involving FGF21 analogs. Other combinations have been investigated primarily in preclinical models. Findings from mouse models of MASH provide mechanistic evidence and may support future clinical investigation, but they should not be presented as evidence that a corresponding combination is currently being evaluated in clinical trials unless an appropriate clinical trial registration number is available. Overall, whether combination therapies provide additive histological or cardiovascular benefits beyond optimized monotherapy remains to be established in appropriately designed clinical trials [135,136].

## 5. Future Perspectives

Despite remarkable advances in the understanding and management of MASLD/MASH, the next phase of therapeutic development should move beyond demonstrating improvement in liver histology toward demonstrating durable modification of the disease process across organs. Current trials frequently prioritize MASH resolution and/or fibrosis improvement, whereas CVD remains the leading cause of mortality in this population. Future studies should therefore be designed around the concept of cardiometabolic–hepatic benefit, incorporating prespecified cardiovascular and renal outcomes alongside hepatic endpoints. Depending on the population and treatment mechanism, relevant outcomes could include MACE, heart failure hospitalization, atrial fibrillation, kidney outcomes, vascular imaging or endothelial biomarkers, and clinically meaningful changes in noninvasive liver disease measures [37,42,137,138,139].

A second priority is to identify which biological pathways are dominant in an individual patient and at what stage of disease. MASLD/MASH is heterogeneous: some individuals are driven predominantly by obesity and insulin resistance, whereas others may have disproportionate lipotoxicity, inflammatory activation, fibrosis progression, or genetically mediated susceptibility. Genetic variants such as PNPLA3 and TM6SF2, together with circulating proteomic, metabolomic, lipidomic, hepatokine, and inflammatory signatures, may help define mechanistic subphenotypes [32,140,141]. Importantly, biomarkers should not be used only to predict fibrosis; their greater future value may lie in predicting treatment response, residual cardiovascular risk, and the likelihood of fibrosis regression or progression.

Noninvasive longitudinal monitoring should increasingly complement baseline biopsy-based assessment. Quantitative measures of liver fat, liver stiffness, fibrosis biomarkers, and circulating molecular signatures could be used repeatedly to determine whether a therapy is suppressing the relevant disease pathway and whether treatment intensity should be escalated. Such an approach could ultimately facilitate adaptive treatment algorithms in which therapy is intensified or modified according to early biological responses rather than relying exclusively on late histological endpoints [1,142,143]. However, prospective studies are still needed to establish validated thresholds for changes in noninvasive biomarkers and determine whether these changes reliably predict histological responses and, ultimately, hard hepatic and cardiovascular outcomes.

Combination therapy is likely to become increasingly important, but it should be driven by mechanistic complementarity rather than by the simple accumulation of agents. One rational model is to establish a metabolic backbone that reduces energy excess, IR, and systemic inflammation and then add a liver-directed agent that addresses residual steatosis, lipotoxicity, or fibrogenesis. Another model is to pair agents in which one mechanism offsets a liability of the other, as illustrated by strategies designed to counteract the hypertriglyceridemia associated with ACC inhibition [126]. Importantly, negative combination trials demonstrate that greater reductions in liver fat do not automatically translate into greater antifibrotic efficacy [131,132]. Future combination trials should therefore be enriched for patients with residual pathway activity, use prespecified mechanistic biomarkers, and test whether combination therapy provides incremental benefit over optimized monotherapy.

Treatment sequencing may be as important as treatment selection. Patients with predominant metabolic dysfunction may benefit from therapies that produce substantial weight loss and improve insulin sensitivity, whereas individuals with established F2–F3 fibrosis may warrant consideration of earlier addition of a liver-directed therapy with demonstrated antifibrotic efficacy [142,144]. Similarly, patients with high ASCVD risk should receive evidence-based management of lipid levels, blood pressure, glycemia, and antithrombotic therapy when clinically indicated, irrespective of whether a liver-directed treatment is being used [142,144]. Future trials should explicitly evaluate these therapeutic sequences and determine whether early control of systemic cardiometabolic drivers enhances subsequent fibrosis regression or reduces cardiovascular events.

Novel therapeutic development should increasingly target the biological nodes that connect metabolic stress to inflammation and fibrosis. These include innate immune pathways such as TLR/NLRP3 signaling, macrophage phenotype and resolution biology, fibrogenic mediators and HSC activation, bile-acid/FXR–FGF19 signaling, and inter-organ communication involving adipose tissue, gut, and liver [32,35,37]. The goal should not simply be to suppress a single inflammatory mediator but to interrupt the feed-forward loops that sustain hepatocyte injury, immune activation, and extracellular-matrix deposition. At the same time, cardiovascular safety must be incorporated early into development programs for agents that alter lipid flux, because an apparently successful reduction in hepatic steatosis may be offset by adverse changes in circulating atherogenic lipoproteins.

Finally, the field should move toward a cardio-hepato-metabolic model of drug development in which hepatic, cardiovascular, renal, and metabolic outcomes are considered as interconnected rather than separate domains. This approach will require multidisciplinary trials involving hepatology, endocrinology, cardiology, and primary care, with harmonized definitions of clinically meaningful benefit and sufficiently long follow-up to detect cardiovascular and liver-related events. Such trials could establish whether improvements in liver histology translate into fewer MACE and heart failure events, whether cardiovascular benefit occurs independently of fibrosis regression, and which patient phenotypes derive the greatest net clinical benefit [6,138]. Ultimately, precision medicine in MASLD/MASH should integrate disease stage, metabolic phenotype, genetic susceptibility, treatment response, comorbidities, and baseline cardiovascular risk to guide individualized treatment intensity and sequencing.

## 6. Conclusions

The evolving understanding of MASLD/MASH supports a fundamental shift from a liver-centered condition toward a systemic cardiometabolic disease in which hepatic, adipose, metabolic, immune, and vascular processes are biologically interconnected. Deeper mechanistic understanding indicates that insulin resistance, substrate overflow, lipotoxicity, mitochondrial and endoplasmic reticulum stress, innate immune activation, fibrogenesis, and endothelial dysfunction form interdependent pathways that can sustain both liver and cardiovascular injury. This framework helps explain why therapies directed at a single disease component may be insufficient for some patients and provides a rationale for mechanism-based combination and sequencing strategies. Future therapeutic development should therefore prioritize patient stratification, longitudinal biomarker-guided monitoring, integrated hepatic–cardiovascular endpoints, and agents capable of interrupting the specific pathways that remain active despite metabolic improvement. A cardio-hepato-metabolic approach, integrating liver-directed therapy with evidence-based cardiovascular risk reduction and multidisciplinary care, may ultimately provide the greatest opportunity to improve long-term clinical outcomes in MASLD/MASH.

## Figures and Tables

**Table 1:** Major clinical trials on emerging therapeutic options targeting MASLD/MASH and cardiovascular risk.

Trial	Drug (Class)/Comparator	Phase	Population/Sample Size/Duration	Main Endpoint(s)	Key Results	Cardiovascular Relevance
NATiVE (NCT03008070) [74]	Lanifibranor (pan-PPAR agonist)/Placebo	Phase 2b	MASH patients/247/24 weeks	MASH resolution and fibrosis improvement	MASH resolution without fibrosis worsening: 22% (placebo) vs. 39% (800 mg) vs. 49% (1200 mg); fibrosis improvement ≥ 1 stage: 29% vs. 34% vs. 48%	PPAR activation improves insulin sensitivity, TG, inflammation, endothelial function
ESSENCE (NCT04822181) [79]	Semaglutide (GLP-1 RA)/Placebo	Phase 3 (ongoing)	MASH with moderate/advanced fibrosis/1197 overall; included in the reported 72-week interim histology analysis/240 weeks total planned	Histological improvement	Interim results showed improved MASH resolution and reduced liver scarring	GLP-1 RAs reduce MACE, improve endothelial function and metabolic risk factors
SYNERGY-NASH (NCT04166773) [81]	Tirzepatide (GLP-1/GIP dual agonist)/Placebo	Phase 2	Biopsy-confirmed MASH, fibrosis F2–F3/190/52 weeks	MASH resolution without fibrosis worsening	Significantly increased MASH resolution vs. placebo	Associated with weight loss and CV risk reduction
LEADER (NCT01179048) [65]	Liraglutide (GLP-1 RA)/Placebo	CV Outcome Trial	high-risk T2DM patients/9340/3.8 years	MACE	Reduced MACE risk by 13%	Demonstrated CV benefit
SUSTAIN-6 (NCT01720446) [88]	Semaglutide (GLP-1 RA)/Placebo	CV Outcome Trial	T2DM with high CV risk/3297/104 weeks	CV death, nonfatal MI, nonfatal stroke	Significant reduction in composite CV outcomes	Strong evidence for CV protection
HARMONY (NCT04767529) [102]	Efruxifermin (FGF21 analog)/Placebo	Phase 2	MASH patients/128/24 weeks	Fibrosis improvement without worsening MASH	Significant fibrosis improvement without worsening of MASH in 41% (50 mg) and 39% (28 mg) vs. 20% in the placebo group	Improved lipids, insulin sensitivity, weight, glycemic control
ENLIVEN (NCT04929483) [103]	Pegozafermin (FGF21 analog)/Placebo	Phase 2b	MASH with F2–F3 fibrosis/222 randomized/24 weeks	Fibrosis improvement without worsening MASH	Fibrosis improvement without worsening of MASH: 22%, 26%, and 27% vs. 7% with placebo, depending on dose	Favorable lipid profile effects
NCT04880031 [104]	Efimosfermin alfa (FGF21 analog)/Placebo	Phase 2a	MASH with fibrosis/84 randomized/24 weeks	Fibrosis improvement and MASH resolution	Fibrosis improvement: 45.2% vs. 20.6%; MASH resolution: 67.7% vs. 29.4% placebo	Potential cardiometabolic benefits under investigation
MAESTRO-NASH (described in article as Phase 3 resmetirom trial) (NCT03900429) [111]	Resmetirom (THR-β agonist)/Placebo	Phase 3	MASH with fibrosis/966/52 weeks	MASH resolution and fibrosis improvement	MASH resolution: 25.9% (80 mg), 29.9% (100 mg) vs. 9.7% placebo; fibrosis improvement: 25% vs. 14.2% placebo	Reduced LDL-C, non-HDL-C, ApoB and triglycerides
NCT02927184 [114]	VK2809 (THR-β agonist)/Placebo	Phase 2	Hypercholesterolemia and MASH or MASH alone/248 actual randomized/52 weeks	Reduction in liver steatosis	~50% achieved steatosis reduction vs. 9% controls	Potential dual hepatic and lipid-lowering benefits
VOYAGE (NCT04173065) [114]	VK2809 (THR-β agonist)/Placebo	Phase 2	Biopsy-proven MASH with F1–F3 fibrosis/248 adults/52 weeks	Relative change in liver fat content from baseline to Week 12, as measured by magnetic resonance imaging proton density fat fraction (MRI-PDFF)	Patients treated with VK2809 achieved statistically significant relative reductions in liver fat through week 52 (56% median reduction from baseline)	Potential hepatic and lipid effects; cardiovascular outcome benefit not established
NCT01431521 [115]	MK-4075 (ACC inhibitor)/Placebo; pioglitazone 30 mg/day was also included as an active comparator	Phase 1	MASLD/MASH patients/31/4 weeks	Reduction in hepatic steatosis	36% reduction in liver fat after 1 month	Limitation: increased triglycerides
PF-05221304 study [116]	PF-05221304 (ACC inhibitor) ± DGAT inhibitor/Placebo	Phase 2a	MASLD/MASH patients/305/16 weeks	DNL inhibition and liver fat reduction	82% DNL reduction; 50–65% steatosis reduction	TG elevation remains concern
MIRNA Study (NCT04321031) [122]	Ervogastat (DGAT2 inhibitor) ± Clesacostat (ACC inhibitor)/Placebo	Phase 2	MASH patients/255/48 weeks	MASH resolution without fibrosis worsening	Primary endpoint comprised MASH resolution without worsening of fibrosis, fibrosis improvement without worsening of MASH, or both; combination demonstrated histological efficacy, whereas ervogastat monotherapy did not meet the composite endpoint	Combination associated with potentially unfavorable lipid and apolipoprotein changes; long-term cardiovascular implications uncertain

ACC: Acetyl-CoA Carboxylase; ApoB: Apolipoprotein B; CV: cardiovascular; DGAT: Diacylglycerol Acyltransferase; DNL: de novo lipogenesis; FGF21: Fibroblast Growth Factor 21; GIP: Gastric Inhibitory Polypeptide; GLP-1: Glucagon-Like Peptide-1; HDL: High-Density Lipoprotein; LDL: Low-Density Lipoprotein; MACE: Major Adverse Cardiac Event; MASH: Metabolic dysfunction-Associated Steatohepatitis; MASLD: Metabolic Dysfunction-Associated Steatotic Liver Disease; MI: Myocardial Infarction; PPAR: Peroxisome Proliferator-Activated Receptors; RA: Receptor Agonist; T2DM: Type 2 Diabetes Mellitus; TG: Triglycerides; THR-β: Thyroid hormone receptor-β.

## Data Availability

The original contributions presented in this study are included in the article. Further inquiries can be directed to the corresponding author.

## Abbreviations

The following abbreviations are used in this manuscript:

ACC	Acetyl-CoA Carboxylase
ApoB	Apolipoprotein B
ASCVD	Atherosclerotic Cardiovascular Disease
CKD	Chronic Kidney Disease
CRP	C-Reactive Protein
CV	Cardiovascular
CVD	Cardiovascular disease
CVOTs	Cardiovascular Outcome Trials
DAG	Diacylglycerol
DGAT	Diacylglycerol Acyltransferase
DNL	de novo lipogenesis
EMA	European Medicines Agency
FASN	Fatty Acid Synthase
FDA	Food and Drug Administration
FFAs	Free Fatty Acids
FGF21	Fibroblast Growth Factor 21
FXR	Farnesoid X receptor
GIP	Gastric Inhibitory Polypeptide
GLP-1	Glucagon-Like Peptide-1
HDL	High-Density Lipoprotein
HSCs	Hepatic Stellate Cells
IL	Interleukin
IR	Insulin Resistance
LDL	Low-Density Lipoprotein
MACE	Major Adverse Cardiac Event
MALO	Major Adverse Liver Outcomes
MASH	Metabolic dysfunction-Associated Steatohepatitis
MASLD	Metabolic Dysfunction-Associated Steatotic Liver Disease
MI	Myocardial Infarction
NAFLD	Non-alcoholic fatty liver disease
NEFAs	Non-esterified fatty acids
NO	Nitric Oxide
PAI-1	Plasminogen Activator Inhibitor-1
PPAR	Peroxisome Proliferator-Activated Receptors
RA	Receptor Agonist
RAAS	Renin–angiotensin–aldosterone system
ROS	Reactive Oxygen Species
SGLT2i	Sodium-Glucose Cotransporter-2 Inhibitors
T2DM	Type 2 Diabetes Mellitus
TGs	Triglycerides
THR-β	Thyroid hormone receptor-β
TNF-α	Tumor Necrosis Factor-α
VLDL	Very Low-Density Lipoprotein
